# Comprehensive Study of the Ammonium Sulfamate–Urea Binary System

**DOI:** 10.3390/molecules28020470

**Published:** 2023-01-04

**Authors:** Aleksandr S. Kazachenko, Noureddine Issaoui, Olga Yu. Fetisova, Yaroslava D. Berezhnaya, Omar M. Al-Dossary, Feride Akman, Naveen Kumar, Leda G. Bousiakou, Anna S. Kazachenko, Vladislav A. Ionin, Evgeniy V. Elsuf’ev, Angelina V. Miroshnikova

**Affiliations:** 1 Department of Organic and Analytical Chemistry, Siberian Federal University, pr. Svobodny 79, 660041 Krasnoyarsk, Russia; 2 Institute of Chemistry and Chemical Technology, Krasnoyarsk Scientific Center, Siberian Branch, Russian Academy of Sciences, Akademgorodok 50, Bld. 24, 660036 Krasnoyarsk, Russia; 3 Department of Biological Chemistry with Courses in Medical, Pharmaceutical and Toxicological Chemistry, Krasnoyarsk State Medical University, St. Partizan Zheleznyak, Bld. 1, 660022 Krasnoyarsk, Russia; 4 Laboratory of Quantum and Statistical Physics (LR18ES18), Faculty of Sciences, University of Monastir, Monastir 5079, Tunisia; 5 Department of Physics and Astronomy, College of Science, King Saud University, P.O. Box 2455, Riyadh 11451, Saudi Arabia; 6 Vocational School of Food, Agriculture and Livestock, University of Bingöl, Bingöl 12000, Turkey; 7 Department of Chemistry, Maharshi Dayanand University, Rohtak 124001, India; 8 IMD Laboratories Co., R&D Section, Lefkippos Technology Park, NCSR Demokritos, P.O. Box 60037, 15130 Athens, Greece

**Keywords:** ammonium sulfamate, urea, density functional theory, binary system, quantum theory of atoms in molecules

## Abstract

The physicochemical properties of binary systems are of great importance for the application of the latter. We report on the investigation of an ammonium sulfamate–urea binary system with different component ratios using a combination of experimental (FTIR, XRD, TGA/DSC, and melting point) and theoretical (DFT, QTAIM, ELF, RDG, ADMP, etc.) techniques. It is shown that, at a temperature of 100 °C, the system under study remains thermally and chemically stable for up to 30 min. It was established using X-ray diffraction analysis that the heating time barely affects the X-ray characteristics of the system. Data on the aggregate states in specified temperature ranges were obtained with thermal analysis and determination of the melting point. The structures of the ammonium sulfamate–urea system with different component ratios were optimized within the density functional theory. The atom-centered density matrix propagation calculation of the ammonium sulfamate–urea system with different component ratios was performed at temperatures of 100, 300, and 500 K. Regardless of the component ratio, a regular increase in the potential energy variation (curve amplitude) with an increase in temperature from 100 to 500 K was found.

## 1. Introduction

Urea (carbamide, diamide of carbonic acid) is a low-molecular-weight hygroscopic organic molecule composed of a carbonyl group attached to two amine residues [1,2]. It is a white crystal soluble in polar solvents (water, ethanol, and liquid ammonia) [3].

Urea is produced with a large capacity for use mainly as a nitrogen-release fertilizer [4,5]. Another important industrial application of urea is the synthesis of urea–formaldehyde resins, which serve as adhesives in fiberboard and furniture production [6,7]. In addition, urea derivatives are known to be efficient herbicides [8,9]. Urea and its derivatives find application in medicine [1,10].

The physicochemical properties of mixtures of urea with various substances are important to investigate in order to establish the origin of intermolecular interactions and understand their interplay [11,12,13].

Ammonium sulfamate is a crystalline ammonium salt of sulfamic acid highly soluble in water [14]. It can be used as a herbicide [15], a fire-protecting additive [16], an additive in the fabrication of cigarette paper [17], and a deicer [18]. The physicochemical properties of ammonium sulfamate are being intensively studied to expand its application range [19,20,21,22].

In [18], the eutectic and other thermodynamic properties of a water–urea–ammonium sulfamate system were experimentally examined. The study was based mainly on thermodynamic modeling and, according to the results of the physicochemical analysis, only the differential scanning calorimetry (DSC) data on the solidus–liquidus point were reported. In [23], the volumetric properties of this system were explored.

Despite the increasing number of works on binary systems in general and urea in particular, the ammonium sulfamate–urea system remains understudied. In this work, the ammonium sulfamate–urea system with different component ratios is characterized experimentally (FTIR, XRD, and TDA/DSC) and theoretically (DFT, QTAIM, RDG, etc.).

## 2. Results and Discussion

### 2.1. Experimental Study of the Ammonium Sulfamate–Urea System

#### 2.1.1. FTIR

**Figure 1:**
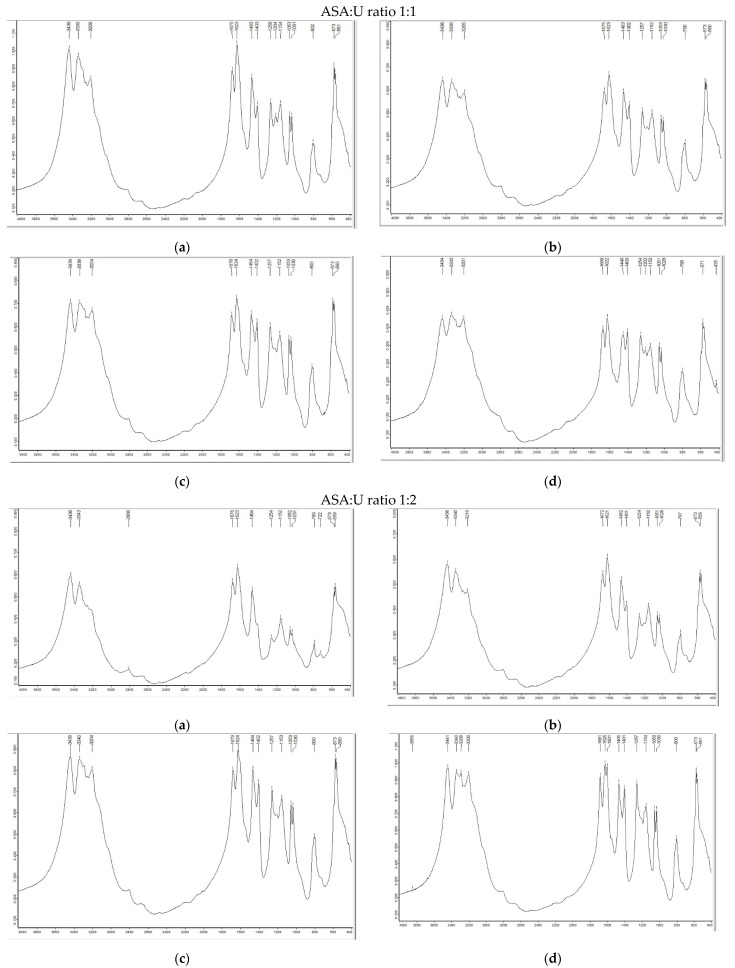

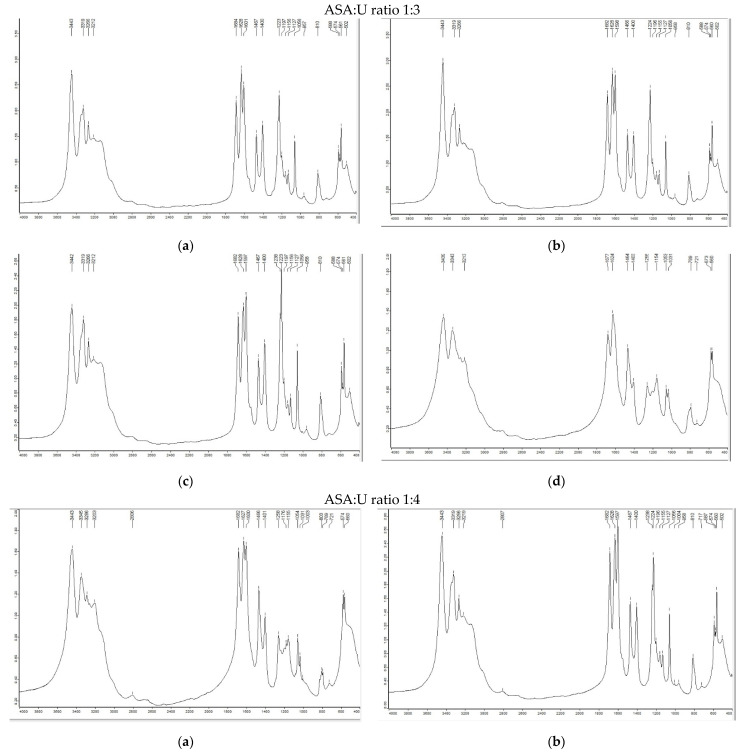

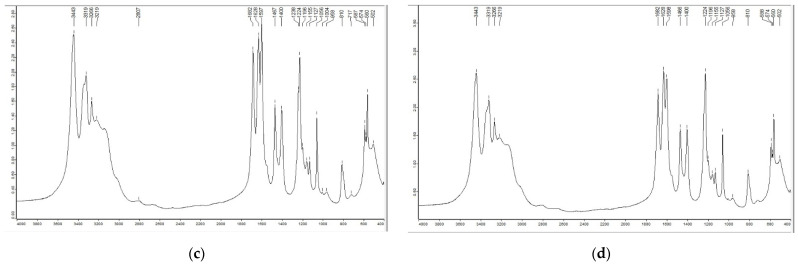
FTIR spectra of the ammonium sulfamate–urea system with different component ratios after heating at 100 °C for (**a**) 0, (**b**) 5, (**c**) 15, and (**d**) 30 min.

The synthesized ammonium sulfamate–urea system with different component ratios was analyzed using infrared (FTIR) spectroscopy (Figure 1).

For all the investigated samples, many absorption bands were observed in the spectra within 3500–2800 cm^−1^, which correspond to the NH_3_^+^ and NH_2_ vibrations [24]. The abundance of absorption bands in the spectra is related to the amino and ammonium groups in ammonium sulfamate and urea, as well as to the effect of different groups on them, which lead to the protonation and deprotonation in the system [25]. The bands at 1250–1260 and 1060–1070 cm^–1^ can be attributed to the superposition of vibrations of the C–N and SO_3_ groups in this region.

To establish the thermal and chemical stability of the ammonium sulfamate–urea system, samples with different component ratios were examined using FTIR spectroscopy after heating at 100 °C for 0–30 min (Figure 1). The data obtained (Figure 1) suggest that the chemical interaction between ammonium sulfamate and urea with the formation of new chemical bonds does not occur. In other words, this mixture is chemically and thermally stable at temperatures of up to 100 °C for up to 30 min. For more detail concerning the thermochemical properties, see Section 2.1.3 (TGA/DSC).

#### 2.1.2. XRD

The XRD analysis is used to identify crystalline phases and determine their relative concentrations in mixtures, unit cell parameters of a known substance for detecting isomorphic impurities, parameters and possible space groups of new compounds, etc. [26,27].

**Figure 2:**
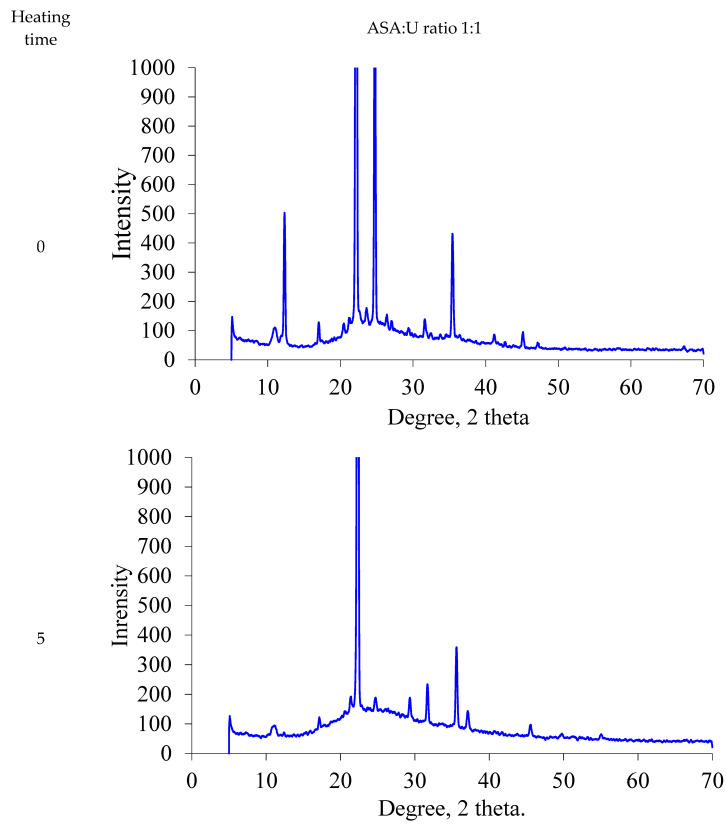

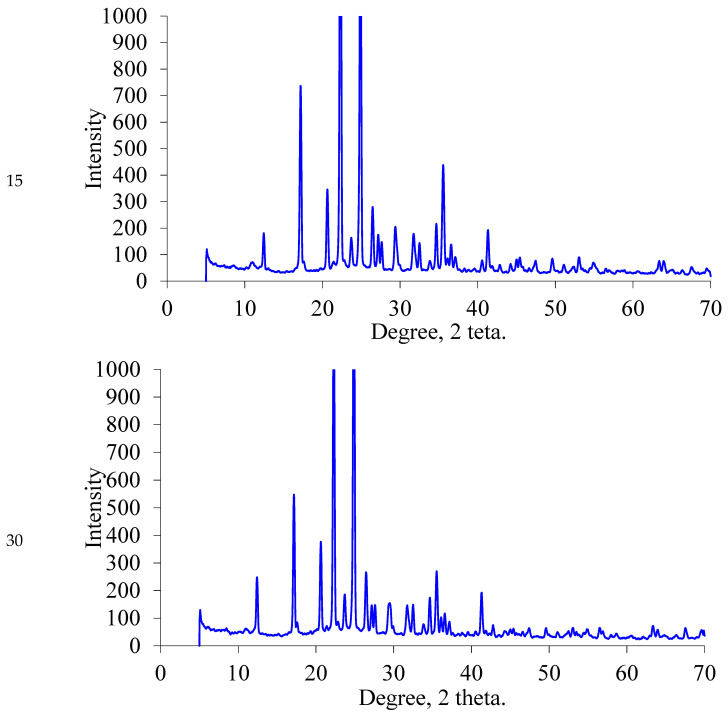

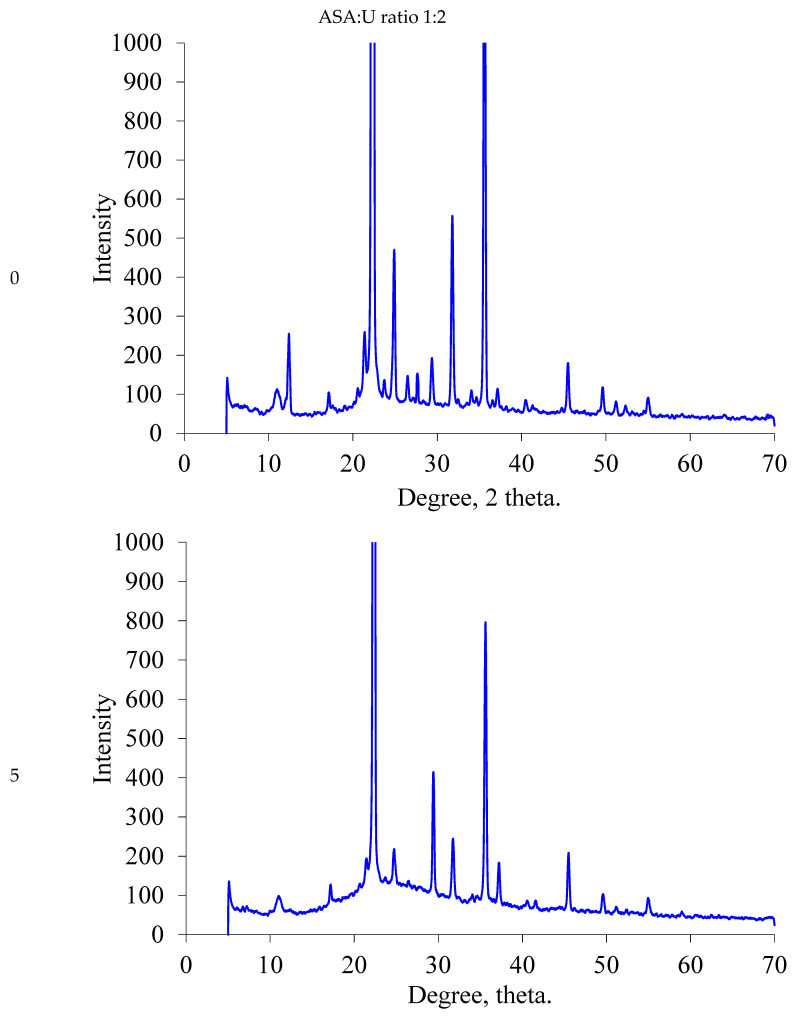

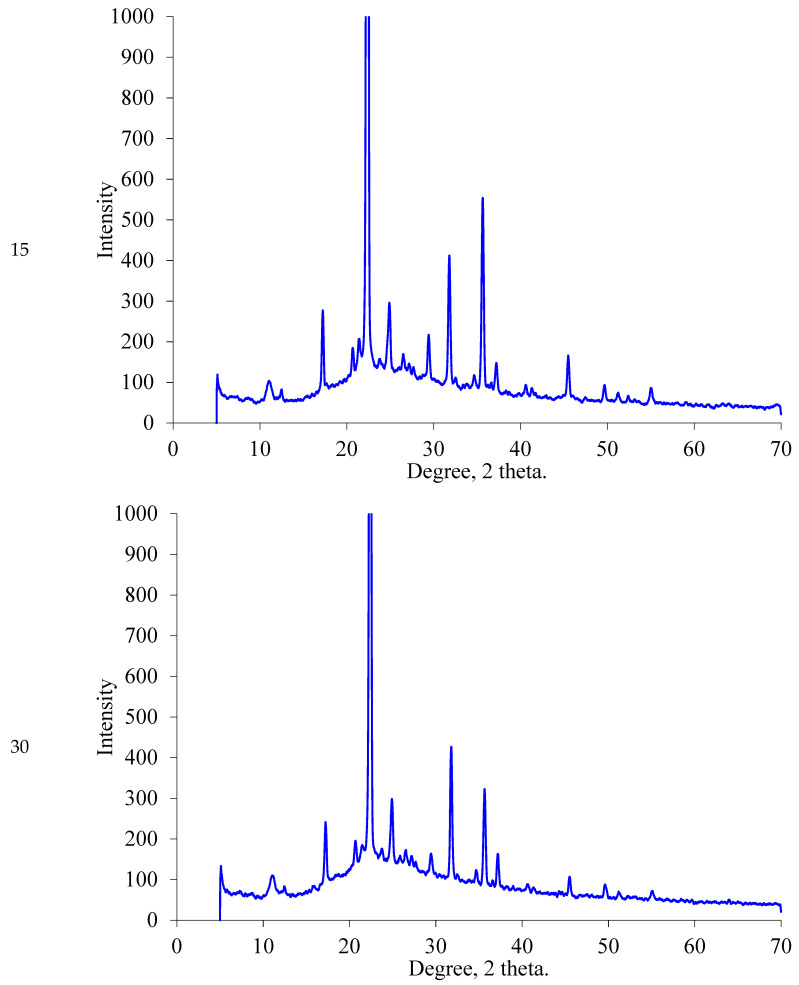

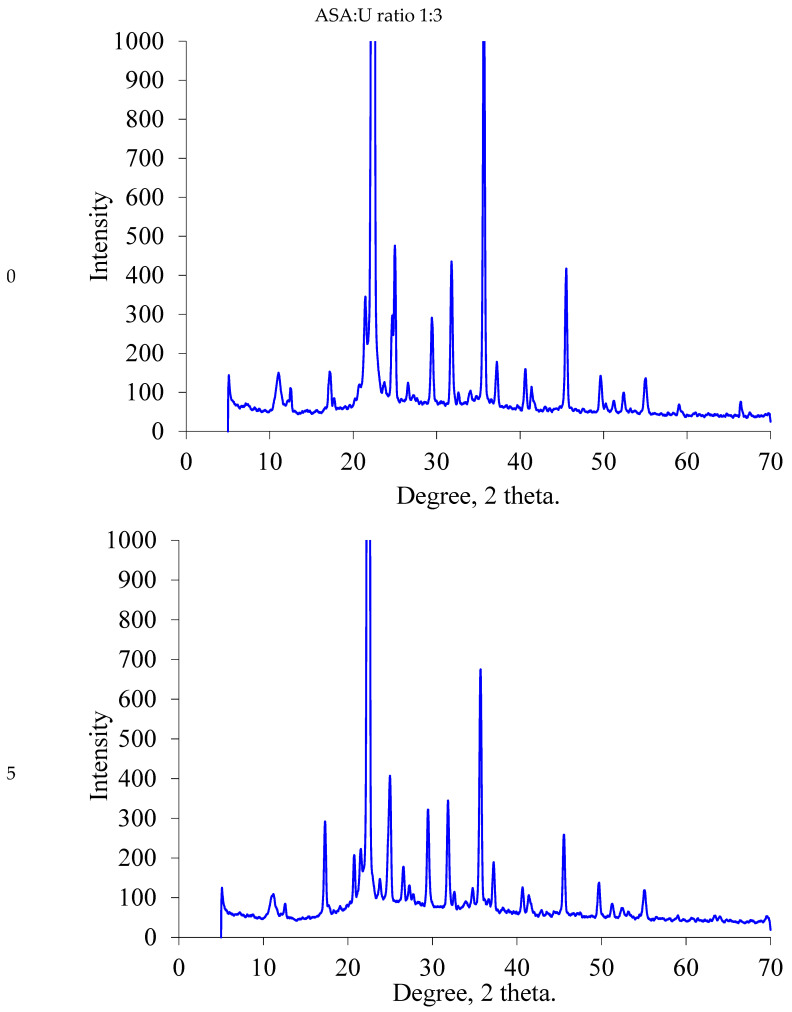

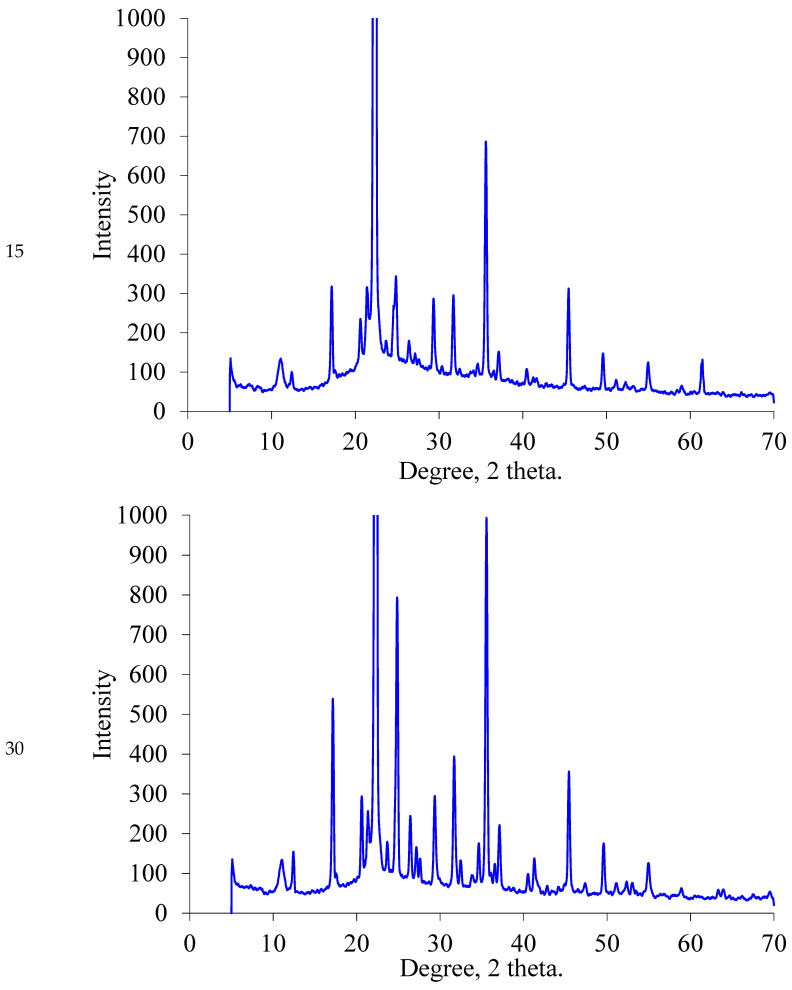

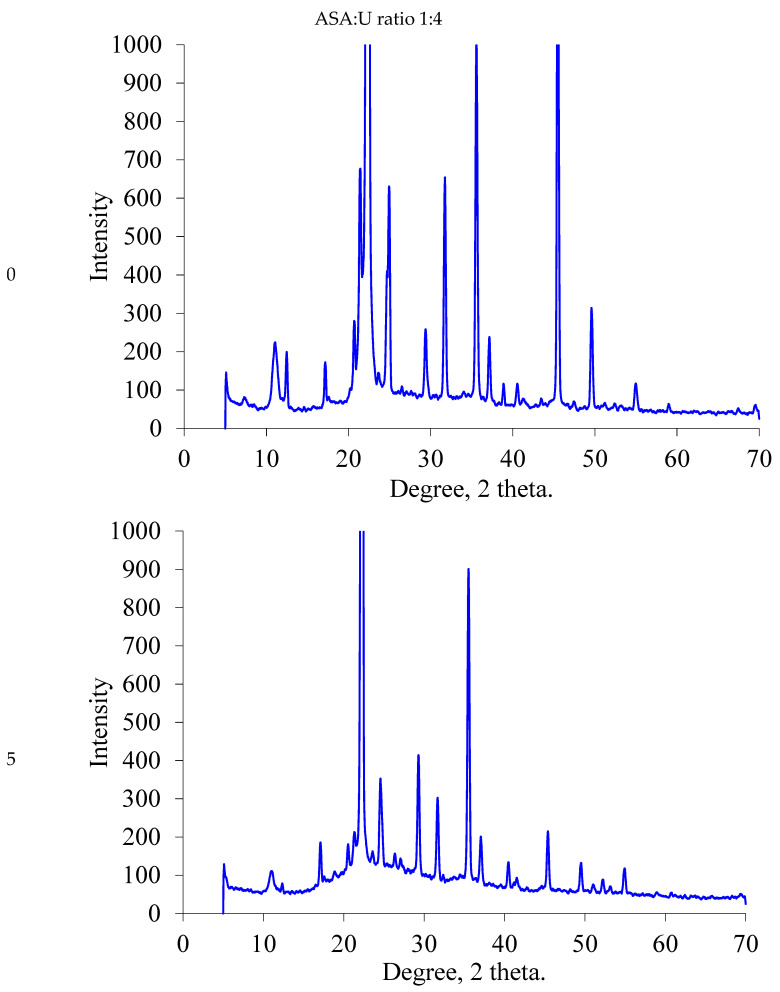

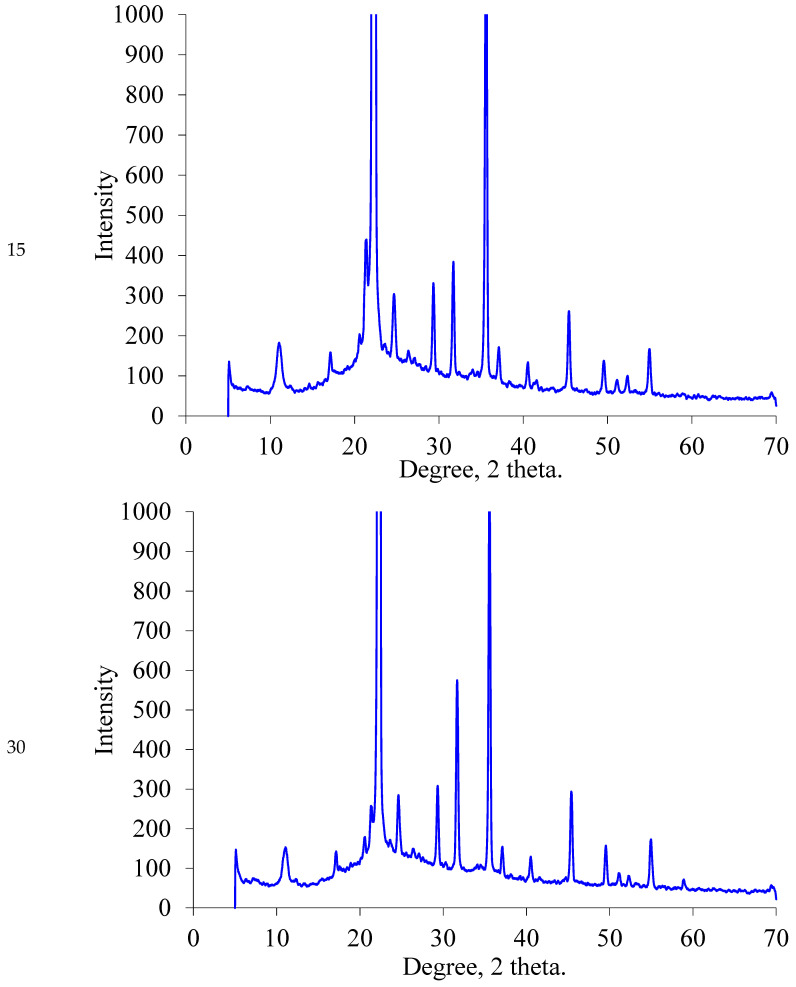
XRD patterns for the ammonium sulfamate–urea system with different component ratios heated for different times.

We carried out the XRD investigations of the ammonium sulfamate–urea samples with different component ratios processed at a temperature of 100 °C for different times (Figure 2).

It can be seen in Figure 2 that a change in the component ratio in the ammonium sulfamate–urea system is reflected in a change in the XRD spectra. In particular, the peaks at 35 and 45° 2θ increase significantly with the urea content in the system.

Heating of the investigated system changes the XRD spectra. In general, in the region of 24–34° 2θ, the intensity of the peaks lowers and they slightly shift. However, this is only observed at certain component ratios. At an ammonium sulfamate: urea ratio of 1:1 and a heating time of 5 min, the material is amorphized; as the processing time increases, its crystallinity returns.

#### 2.1.3. TGA/DSC

**Figure 3:**
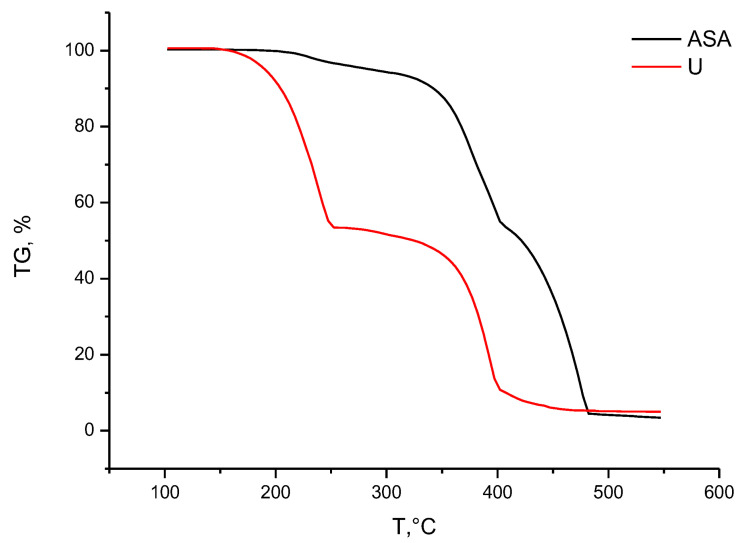
Thermogravimetric analysis of ammonius sulfamate (ASA) and Urea (U).

As can be seen from Figure 3, the thermal decomposition of urea occurs in two stages: 150–252 °C (decomposition of the acid itself with the release of ammonia) and 252–402 °C (decomposition of the resulting isocyanic acid) [28].

The main decomposition of ammonium sulfamate proceeds almost in one stage with a slight inflection at 402 °C. The main decomposition takes place in the range of 317–482 °C.

**Figure 4:**
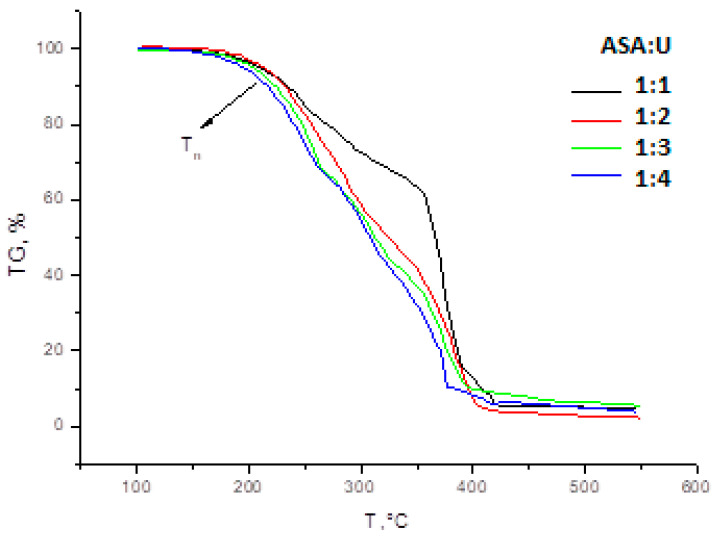
Results of thermogravimetric analysis (TG) of a mixture of ammonium sulfamate with urea.

Figure 4 presents the results of thermogravimetric analysis (TG) of a mixture of ammonium sulfamate with urea with different ratios of components.

The TG curve of sample 1:1 forms a clear inflection at a temperature of 355 °C.

In the series 1:1–1:2–1:3–1:4, the temperature of the beginning of decomposition of the substance (Tn) decreases: 175–175–160–146 °C, along with the interval of complete thermal decomposition from 250 to 230 °C.

#### 2.1.4. Kinetic Analysis of the Thermal Decomposition of the Ammonium Sulfamate–Urea System

Theoretical calculations of the decomposition of the ammonium sulfamate-urea system were carried out using the Coates–Redfern kinetic model [29]. The kinetics of decomposition of the studied samples in the region of the main thermal destruction of the substance was analyzed (TGA data). The results are presented in Table 1.

As can be seen from the data in Table 1, with an increase in the content of urea molecules in the mixture to 2, the activation energy of the thermal destruction of the mixture decreases, while the pre-exponential factor increases, which reflects the frequency of collisions of active molecules. These characteristics give reason to believe that with this increase, the thermal stability of the mixture decreases (the number of less reactive interactions increases).

At a ratio of 3, the activation energy slightly decreased, and with an increase in the ratio of components to 4, the value of the activation energy remained at the same level. Thus, it can be assumed that for samples 3 and 4, in the period of the main thermal decomposition, the bonds of groups with similar properties break.

The activation energy of the decomposition of ammonium sulfamate (49.6 kJ/mol) is higher than the activation energy of sulfamic acid (46.8 kJ/mol) [30]. This also affects the values of pre-exponential factors: for ammonium sulfamate −7.2 × 10^6^ s^−1^, and for sulfamic acid −2.1 × 10^5^ s^−1^. In addition, these differences also affect their mixtures with urea. For mixtures of ammonium sulfamate with urea in ratios of 1:1, 1:2, 1:3, and 1:4, activation energies are observed: 54.7, 48.5, 45.5, and 45.4 kJ/mol, respectively. For a mixture of sulfamic acid with urea with similar molar ratios, the following activation energies are observed: 58.0, 53.4, 47.6, and 44.8 kJ/mol, respectively [30]. The values in these systems are insignificant due to the proximity of the structure of these substances.

#### 2.1.5. Melting Point and Thermodynamic Calculation

The melting point is an important characteristic of binary systems, including crystalline ones [31]. In the ammonium sulfamate–urea system with different component ratios, the melting point of the mixture is generally lower than that of the initial materials (see Table 2) due to the eutectic phenomena in the mixture. The ammonium sulfamate–urea system with a component ratio of 1:1 has the lowest melting point.

Compared to the sulfamic acid-urea system [30], higher melting points are observed for similar ratios (Table 2).

Based on the data on the melting points and results of the thermal analysis, we generalized the temperature dependence of the state of aggregation of the ammonium sulfamate–urea mixture with different component ratios (Table 3).

It should be noted that there is a certain error in the data obtained, which is composed from the instrumental error and the sample humidity influencing these characteristics.

The melting points obtained were used to calculate the physicochemical parameters, including the chemical potential and effective interaction parameter. In the calculation, it was assumed that the ammonium sulfamate–urea system is similar in some physicochemical properties to the sulfamic acid–urea system, which is a deep eutectic solvent [30]. Further calculation of the characteristics is justified by comparing these two systems.

The Δμ_i_^T^ values calculated using Equation (1) are given in Table 4.

According to the data given in Table 3, the Δμ_i_^T^ value for ammonium sulfamate in the ammonium sulfamate–urea system ranges from −2.06 to −4.94 kJ/mol. For urea in this system, the Δμ_i_^T^ values are from −0.68 to −2.06 kJ/mol. In general, this parameter for the ammonium sulfamate–urea system is comparable with that for the sulfamic acid–urea system [30], differing only at some points.

The effective interaction parameter χ calculated directly from the melting point of the mixture makes it possible to reliably describe the complete solid–liquid equilibrium in the system [32].

The effective interaction parameter ranges from −2.46 to −2.77 for ammonium sulfamate and from −2.77 to −5.57 for urea (Table 5). With an increase in the number of urea molecules in the ammonium sulfamate–urea system, the effective interaction parameter decreases from −2.77 to −5.57. In general, the effective interaction parameter χ of the ammonium sulfamate–urea system is comparable to that of the sulfamic acid–urea deep eutectic solvent [30].

According to the data in Table 4, the strong attraction between molecules in the ammonium sulfamate–urea binary mixture is reflected in the effective interaction parameter. It is known well [32] that the strong attraction between components in a system can reduce the melting point. Hydrogen bonds between components in a mixture can also affect this parameter. To study the molecular structure and electronic configuration in more detail, we carried out the quantum-chemical examination.

### 2.2. Theoretical Study of the Ammonium Sulfamate–Urea System

Previously [30], we studied the mixture of sulfamic acid with urea, which represents a deep eutectic solvent. There is no exact information in the literature on whether the ammonium sulfamate–urea mixture is a eutectic solvent. Nevertheless, study of the interaction between these substances can be relevant for understanding its nature and determining the range of possible applications of the mixture.

Quantum-chemical research methods are actively used to study binary systems [33,34]. For small molecules, the DFT method in the B3LYP/6-311++G(d, p) basis set showed the highest efficiency [35,36].

**Figure 5:**
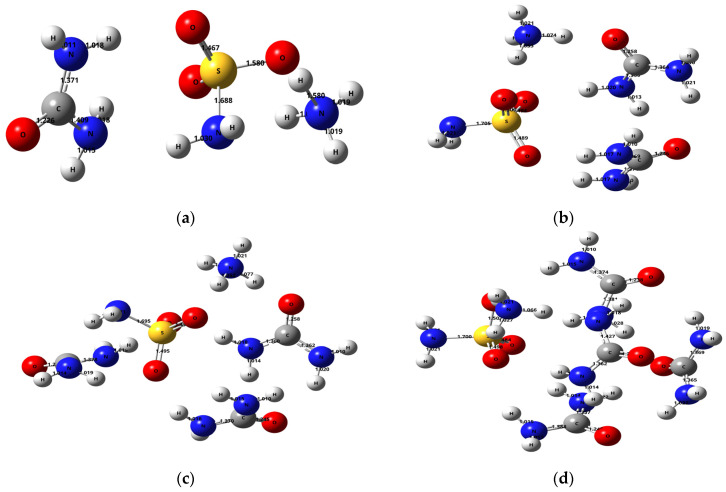
Optimized structures of the ammonium sulfamate system with (**a**) one, (**b**) two, (**c**) three, and (**d**) four urea molecules.

The primary optimization of the structure of the ammonium sulfamate–urea system with different component ratios was carried out using the DFT method in the B3LYP/6-311++G(d, p) basis set (Figure 5).

It can be seen in Figure 5 that the change in the component ratios in the ammonium sulfamate–urea system leads to a change in the ammonium sulfamate and urea bond lengths. In particular, the N–H bond lengths in the ammonium cation of the ammonium sulfamate–urea system with one urea molecule range within 1.019–1.580 Å (Figure 5a). When the second urea molecule is added to the system, this range changes for 1.021–1.074 Å (Figure 5b). In addition, it was observed that the addition of more urea molecules had little effect on the ammonium cation bond lengths in the studied system. The bond length for a sulfo group of ammonium sulfamate (S=O) in the system with one urea molecule is 1.467 Å. One more urea molecule added elongates this bond to 1.489 Å. Further addition of urea molecules changes this bond length insignificantly.

For the S–N bond in ammonium sulfamate, lengths of 1.688–1.700 Å were observed. It is worth noting that, in the sulfamic acid–urea system studied in [30], the length of this bond decreased from 1.6658 to 1.6117 Å with an increase in the number of urea molecules in the system from one to four. At the same time, the length of this bond in aqueous ammonium sulfamate clusters [22] ranges from 1.6851 to 1.6975 Å, while with an increase in the number of water molecules from one to four, the S–N bond extends. Despite the data obtained, it has not been explained by a quantum-chemical approach yet why urea is a less efficient activator when arabinogalactan is sulfated with ammonium sulfamate [37] than when it is sulfated with sulfamic acid [38].

#### 2.2.1. HOMO-LUMO Analysis

The FMO theory, including the highest occupied and lowest unoccupied molecular orbitals (HOMO-LUMO) is one of the best theories for explaining the chemical stability of molecules [39]. The HOMO and LUMO energies for ligands and complexes provide information on the energy distribution and energy behavior. Negative *E*_HOMO_ and *E*_LUMO_ values are indicative of the stability of compounds [40]. The difference between energies of LUMO and HOMO orbitals, that is, the energy gap (E_LUMO_-E_HOMO_), is indicative of the chemical reactivity and kinetic stability of molecules [41,42]: a molecule with a wide HOMO-LUMO gap is identified as having high chemical hardness and good stability, and is much less polarizable. Molecules with a narrow HOMO-LUMO gap have good chemical reactivity and chemical softness and are more polarizable. That is, a wide HOMO-LUMO gap suggests a high molecular stability and low chemical reactivity [43]. The HOMO and LUMO energies reflect the ability to donate and receive an electron, respectively. A high HOMO energy is characteristic of a molecule more active in reactions with electrophiles, while a low LUMO energy corresponds to molecular reactions with nucleophiles [44]. A molecule with a narrow boundary orbital gap is highly polarizable and exhibits a significant degree of intramolecular charge transfer from an electron donor to an electron acceptor and conjugation, which can affect the biological activity of a molecule [45].

**Figure 6:**
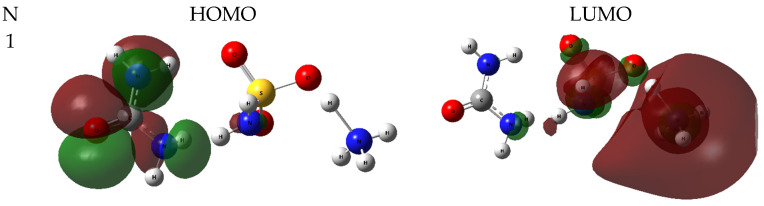

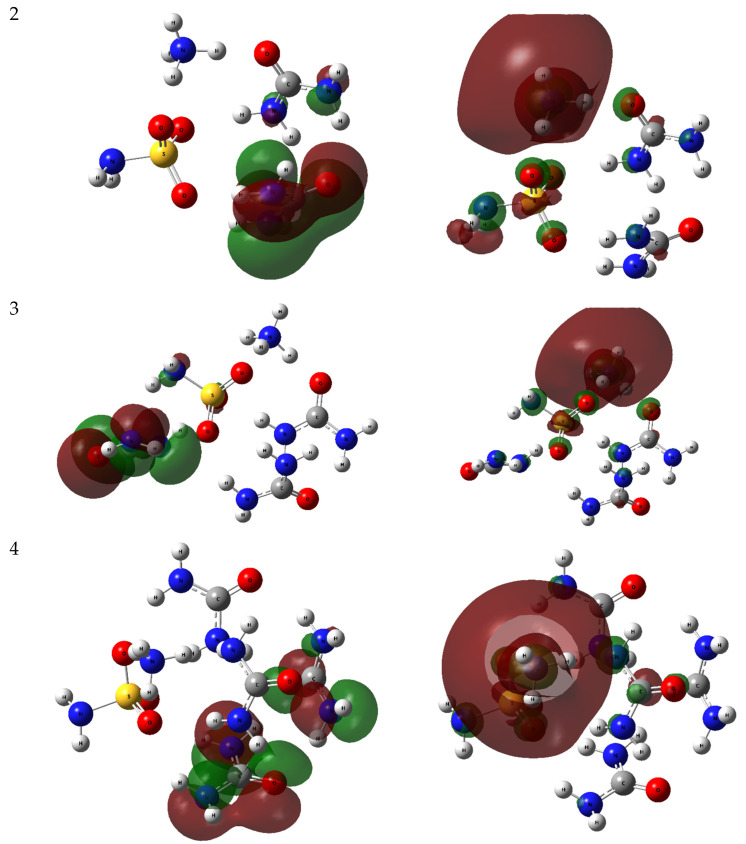
Frontier molecular orbitals in the ammonium sulfamate–urea system with different contents of urea molecules.

A three-dimensional view of HOMO-LUMO is presented in Figure 6.

According to Figure 6 and Table 6, the energy gaps change nonuniformly in the ammonium sulfamate–urea system with an increasing amount of urea. Thus, when another molecule is added to a system with one urea molecule, the energy gap increases from 6.42 to 6.50 eV. With a further increase in the number of urea molecules in the system to four, the energy gap decreases to 5.78 eV. It should be noted that the picture observed for the sulfamic acid–urea system [30] was somewhat different: the energy gap decreased uniformly from 7.39 to 6.69 eV with an increase in the number of urea molecules from one to four. At the same time, the energy gap in water clusters of ammonium sulfamate [22] for a system with one to four water molecules ranges within 7.71–6.68 eV.

We note that the minimum electron affinity, electronegativity, chemical potential, chemical softness, and global electrophilicity index and the maximum charge transfer index, optical softness, ionization potential, chemical hardness, and nucleophilicity index are characteristic of the ammonium sulfamate–urea system containing two urea molecules. The maximum electron affinity, electronegativity, chemical potential, chemical softness, global electrophilicity index, charge transfer index, and optical softness and the minimum ionization potential, chemical hardness, and nucleophilicity index among the investigated systems were observed in the ammonium sulfamate–urea system with four urea molecules.

The calculation of the thermodynamic characteristics is widely used to predict the reactivity and other physicochemical characteristics of compounds [46,47]. Table 7 gives some thermodynamic parameters of the ammonium sulfamate–urea systems with different contents of urea molecules.

According to the data listed in Table 7, the E(RB3LYP) value changes almost uniformly from −962.23145 to −1638.1316 a.u. when the content of urea molecules in the ammonium sulfamate–urea system changes from one to four. The dipole moment, in turn, changes nonuniformly. Thus, a decrease from 7.65 (in the system with one urea molecule) to 5.30 Debye (in the system with two urea molecules) is observed, after which the dipole moment increases to 11.87 Debye (in the system with four urea molecules).

The values of other nonlinear-optical (NLO) parameters, including the hyperpolarizability and polarizability, also increase almost uniformly with the number of molecules in the ammonium sulfamate–urea system. This phenomenon was also observed in the sulfamic acid–urea [30], ammonium sulfamate–water [22], sulfamic acid–water [48], and thiourea–water systems [49].

The correction factors (the zero-point energy correction, thermal energy correction, thermal enthalpy correction, and thermal free energy correction) also increase with the number of urea molecules in the system (see Table 7).

It is noteworthy that the values of the other thermodynamic parameters (E (thermal), heat capacity, and entropy) also increase with the number of urea molecules in the system under study and this increase is almost linear. The results obtained are in good agreement with the data reported in [22,30].

#### 2.2.2. MEP Analysis

In [50,51,52], the MEP was considered as a fundamental factor that determines the nature and behavior of atoms and molecules and their ability for intermolecular interactions. In studies [53,54] aimed at finding the interplay between the experimentally observed properties and the electronic characteristics of particles obtained in the quantum-chemical calculation, the MEP properties were successfully used. The electrostatic potential map is plotted, as a rule, on the particle surface corresponding to an electron density contour close to the boundaries specified by the van der Waals radii. Local values of the total electrostatic potential are used to describe the properties, including the potential ability of particles to be involved in intermolecular interactions.

The MEP analysis is related to the electron density and allows one to establish positions of the electrophilic and nucleophilic attacks and hydrogen bond interactions [55].

**Figure 7:**
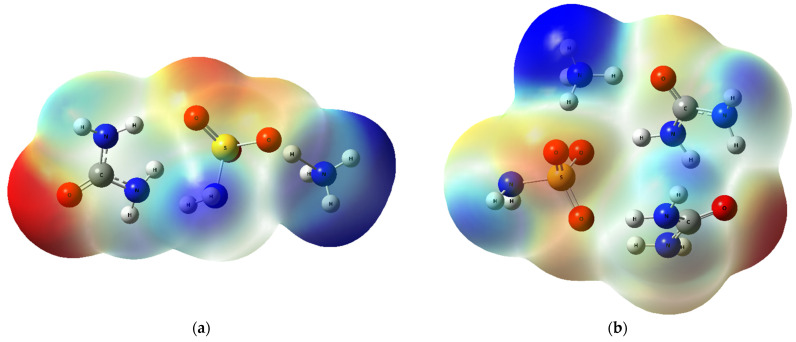

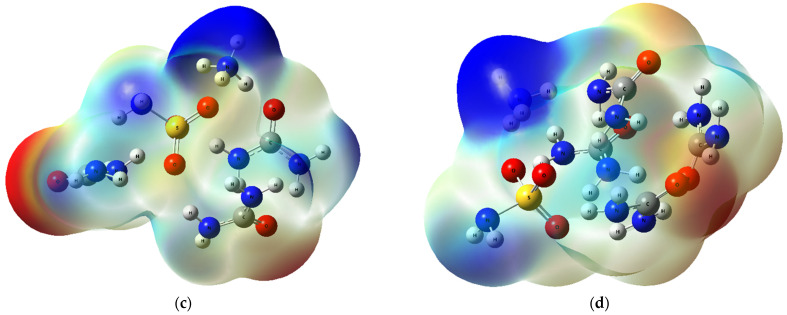
MEP surfaces for (**a**) ASA–urea 1, (**b**) ASA–urea 2, (**c**) ASA–urea 3, and (**d**) ASA–urea 4.

**Figure 8:**
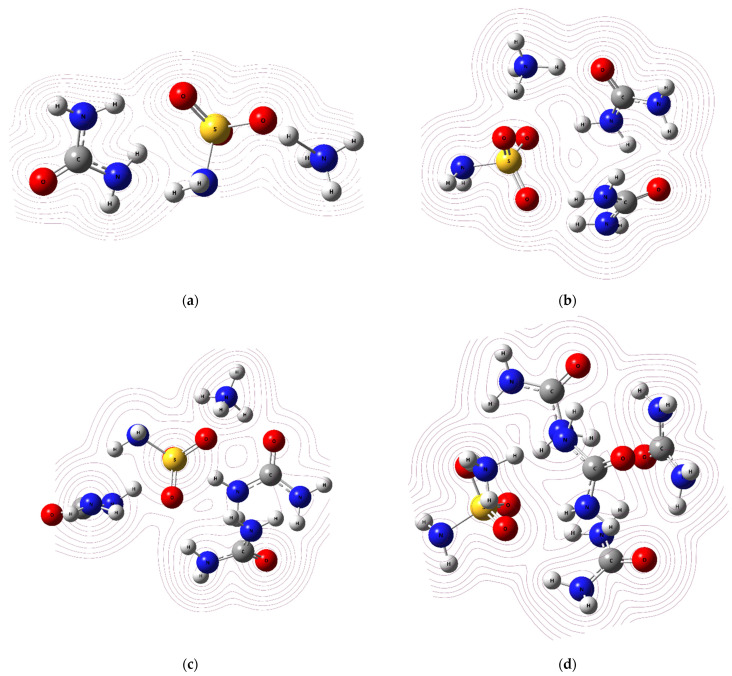
MEP contour maps for (**a**) ASA–urea1, (**b**) ASA–urea2, (**c**) ASA–urea3, and (**d**) ASA–urea4.

The obtained MEP and its contours are shown in Figure 7 and Figure 8, respectively.

In Figure 7, the electrophilic attack region is colored in red (located on oxygen atoms) and the nucleophilic attack region is colored in blue (located on the hydrogens attached to the nitrogen atoms). The size of both regions increases with the number of urea molecules in the ammonium sulfamate–urea system.

#### 2.2.3. QTAIM, ELF, and RDG Analysis

The QTAIM began as an energy distribution theory, which interpreted chemical bonding as a result of the interaction of mononuclear parts. The latter are topological atoms, which, in terms of the electron density topology, are subspaces with a well-defined intrinsic kinetic energy [56,57,58].

**Figure 9:**
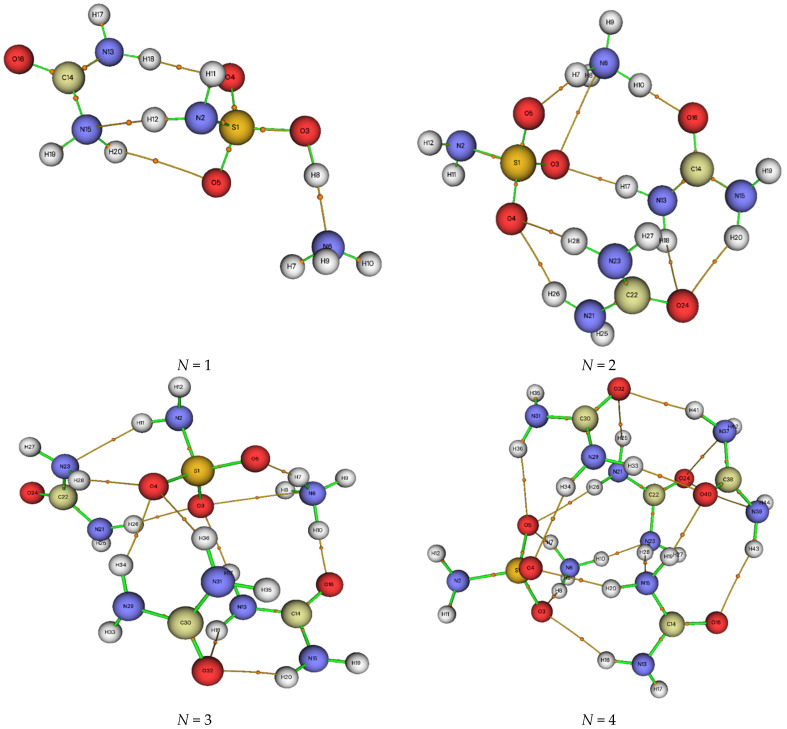
AIM graphs calculated for the compounds with different urea contents.

We carried out the QTAIM calculation for the ammonium sulfamate–urea system with different component ratios (Figure 9, Table S1).

The AIM topological parameters of the ammonium sulfamate–urea system with different contents of urea molecules are listed in Table S1.

The topological parameters of the ammonium sulfamate–urea system with different contents of urea molecules are also given in Table S1 (Supplementary Material). According to these data, at an ammonium sulfamate: urea ratio of 1:1, the observed interactions are H5 … H20, H12 … N15, and O4 … H18 with respective energies of −8.3798, −17.6683, and −20.1578 kJ/mol. At an ammonium sulfamate: urea ratio of 1:2, the observed interactions are H18… O24, O3 … N6, O4 … H28, O4… H26, H20… O24, O3 … H17, O5 … H7, and H10 … O16 with respective energies of −12.1953, −11.1436, −18.1896, −20.8871, −21.9869, −26.195, −49.8004, and −66.7212 kJ/mol. At an ammonium sulfamate: urea ratio of 1:3, the observed interactions are O5 … H7, O3 … H26, O3 … H17, H20 … O32, O4 … H34, O4 … H28, H11 … N23, H18 … O32, O4 … H36, and O3 … N6 with respective energies of −47.7516, −23.6886, −23.4663, −21.528, −20.1968, −15.7976, −14.0075, −13.1014, −12.6802, and −9.2924 kJ/mol. At an ammonium sulfamate: urea ratio of 1:4, the observed interactions are O3 … H8, O5 … H7, O3 … H18, N15 … H28, O4 … H20, O5 … H26, H10 … N23, N23 … N39, O4 … H34, H25 … O32, O5 … H36, O24… N37, O32 … H41, O16 … H43, and O24 … N39 with respective energies of −48.1494, −16.7362, −11.2606, −23.2453, −19.89, −14.8629, −44.6875, −2.7313, −12.181, −8.3252, −10.4897, −3.8675, −22.0675, −15.1177, and −3.0225 kJ/mol.

In general, as compared with the data from [30], more intermolecular interactions were revealed by the QTAIM calculation because of the presence of an additional ammonium cation. At the same time, the interaction energy is of the same order of magnitude and ranges from −3 to −67 kJ/mol, which allows us to speak about the similar nature of the interaction.

The stability of a molecular structure can be aided by molecular interactions; weak intra- or intermolecular interactions can be determined using the RDG calculation based on the analysis of noncovalent interactions (NCIs) [59].

The visual approach known as the RDG analysis is used to make the NCIs more specific and explore intra- and inter-molecular interactions in a molecular system [60]. The RDG function is a fundamental dimensionless quantity for describing the deviation from the uniform electron distribution, which is calculated using the formula [61].

(1) RDG(r)=12(3π2)1/3|∇ρ(r)|ρ(r)43

The RDG scatter plot versus sign of the second eigenvalue (λ_2_)ρ of the electron density provides information about the strength and nature of the interactions. The (λ_2_)ρ value and sign are used to explain the nature of the interactions; (λ_2_)ρ > 0 corresponds to the repulsive interaction and (λ_2_)ρ < 0, to the attractive ones; in the case of the van der Waals interactions, (λ_2_)ρ is close to zero [62].

**Figure 10:**
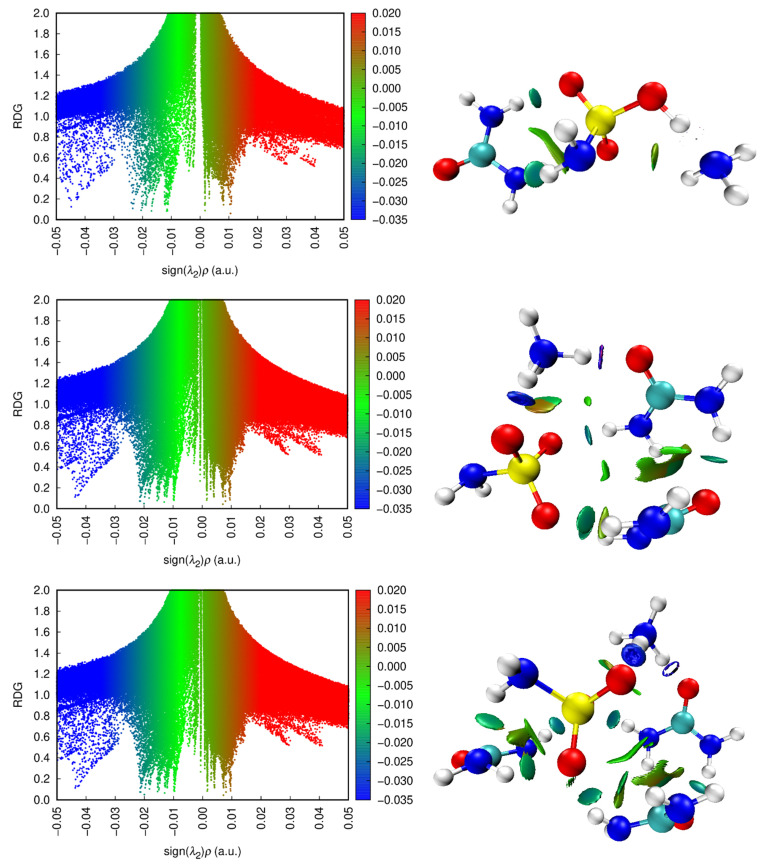

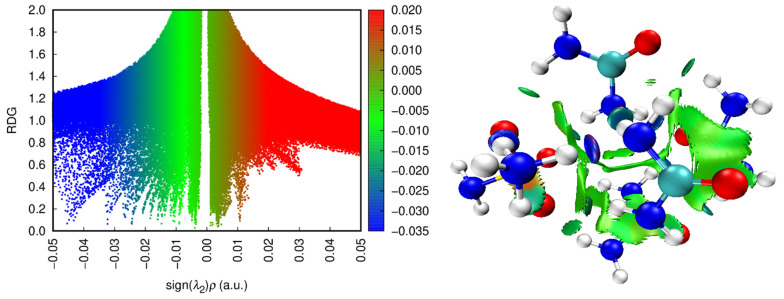
RDG scatter (**left**) and NCI (**right**) plots for the investigated system.

The gradient scatter graphs (RDG) and non-covalent interactions (NCI) for the investigated system are plotted in Figure 10.

The λ_2_(r) function ranges from −0.030 to 0.020 a.u. in the RDG scattering spectra, which are divided into three colors: red, green, and blue. The red peak on the RDG isosurfaces shows the steric repulsion. The RDG scatter plot contains a red contour between 0.02 and 0.05 a.u., which points out the higher contribution of the repulsive exchange. The blue peak is indicative of the strong attraction between the ammonium cation and the urea molecule or the ammonium cation and the sulfo group of ammonium sulfamate and the formation of intermolecular hydrogen bonds. The blue contour in the RDG scatterplot between −0.05 and −0.03 a.u. points out the presence of a hydrogen bond, which is confirmed also by the effective interaction parameter χ. It should be noted that, with an increase in the number of urea molecules, the blue region on the RDG isosurfaces grows. The mixed red-green peaks are observed near the interactions of the ammonium cation with the secondary oxygen atom of the sulfo group of ammonium sulfamate.

**Figure 11:**
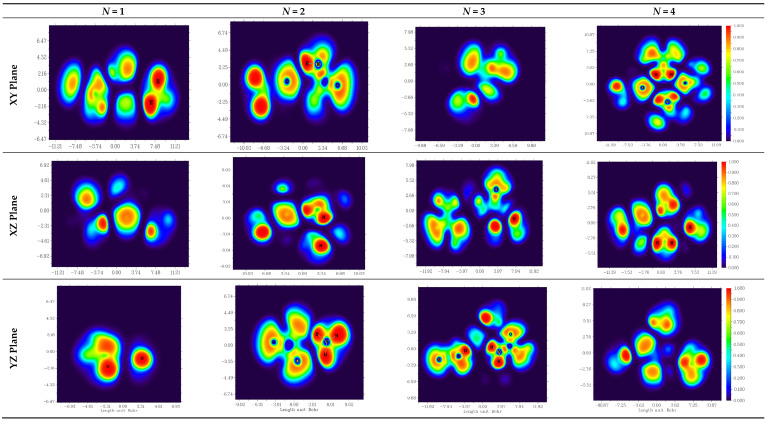
ELF colored maps for the investigated system.

The ELF maps highlight zones of molecular space; the maps of their color shades are shown in Figure 11. This topological surface analysis is based on covalent bonds, in which the electron pair detection is high. The ELF calculation takes into account the kinetic energy density [63,64].

The ELF value τ(r) ranges from 0.0 to 1.0; relatively full values of 0.5 and 1.0 correspond to the contours containing localized bonding and nonbonding electrons [62]. The low (<0.5) values correspond to the contours in which electrons should be delocalized [65]. Figure 11 shows the high ELF regions around hydrogen atoms, which are indicative of the presence of highly localized bonding and nonbonding electrons, and the blue regions around several oxygen and nitrogen atoms show a delocalized electron cloud around them.

### 2.3. Theoretical Spectroscopy Analysis

Raman spectroscopy provides an insight into the structure of a material and its characteristics. It is based on studying scattered light, whereas FTIR spectroscopy is based on the absorption of light [66]. Raman spectroscopy is used to explore intra- and intermolecular vibrations and gives a better idea of the reaction. Both Raman and FTIR spectroscopy are tools for the spectral characterization of molecular vibrations and used to identify substances. At the same time, Raman spectroscopy can yield additional data on the low-frequency modes and vibrations, which reveal features of the crystal lattice and molecular structure [67,68].

#### 2.3.1. NH Bond Vibrations

**Figure 12:**
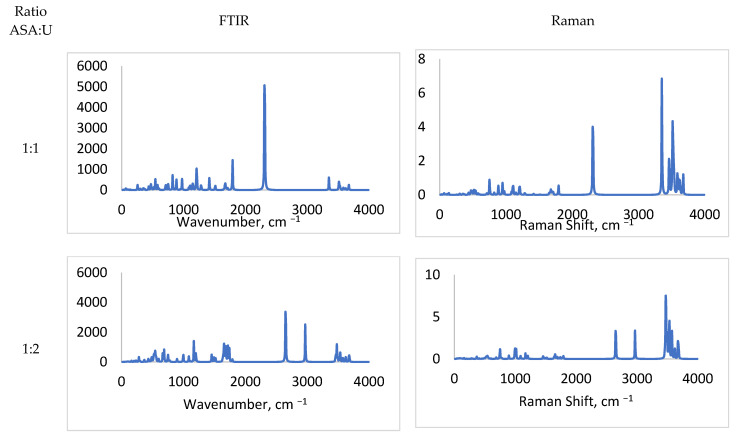

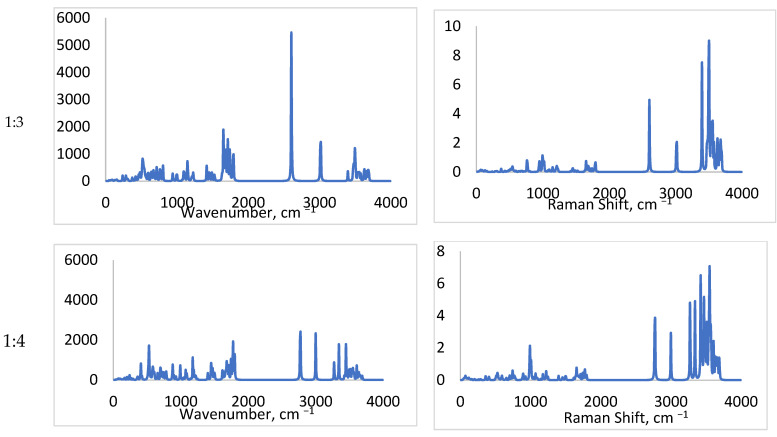
Vibrational spectra of the ammonium sulfamate–urea system with different component ratios.

All the theoretical vibrational FTIR spectra (Figure 12) of the ammonium sulfamate–urea system with different component ratios contain absorption bands of the NH bond in the region of 3200–3600 cm^−1^. It should be noted that the intensity and number of the peaks in this region increase with the content of urea molecules and, consequently, NH_2_ groups and their intermolecular interaction. The Raman spectra yield a similar pattern. The asymmetric vibrations of this group are observed also at 1150–1190 cm^−1^.

#### 2.3.2. SO Bond Vibrations

The SO bond vibrations are observed in the vibrational spectra in the region of 1219–1280 cm^−1^. The introduction of additional urea molecules reduces the vibration intensity of this group and shifts the absorption bands to the lower values.

#### 2.3.3. CO Bond Vibrations

As is known [25], the C=O bond in urea is longer than that found conventionally in the carbonyl group, while the C–N bond is shorter than in amines and amides. The CO bond vibrations are observed in the vibrational spectra of the ammonium sulfamate–urea system at 1600–1700 cm^−1^. The abundance of absorption bands in this region can be related both to the different natures of the interaction between different urea molecules with ammonium sulfamate and to the different states of the CO bond. According to [25], a proton in the urea molecule can migrate from the oxygen to the nitrogen atom. Thus, different forms of urea can be reflected in the abundance of absorption bands for the vibration of one bond in the vibrational spectra.

#### 2.3.4. SN and CN Bond Vibrations

In the FTIR spectra of the ammonium sulfamate–urea system, vibrations of the S–N group are observed in the region of 580–800 cm^−1^, which corresponds to vibrations in some sulfamates and sulfamic acid [69].

The C–N bond vibrations are observed at 1426–1480 cm^−1^ and characteristic of this bond in urea [25].

It should be emphasized that the conventional frequencies of C–N stretching vibrations in aliphatic amines are 1220–1010 cm^−1^ [70]. The band is shifted toward higher frequencies approaching C=N (1690–1640 cm^−1^ [70]), where the CO-group peaks overlap, indicating the transfer of a proton from oxygen to urea nitrogen. The absorption band observed at about 1000 cm^−1^ also belongs to the C–N bond vibration.

### 2.4. ADMP Molecular Dynamic Calculations

Molecular modeling of binary systems is actively used to study the features of these systems [71,72].

To study the stability of the ammonium sulfamate–urea system and the effect of temperature on it, we carried out the molecular dynamics calculation of the atom-centered density matrix propagation (ADMP). The ADMP technique is a special case of an extended Lagrangian molecular dynamics method, which uses the Gaussian basis functions with atom centering and single-particle density matrix propagation [73,74].

The ADMP method is used to study the dynamics of chemical systems. In this approach, various systems can be modeled using all electrons or pseudopotentials based on a DFT code with linear scaling and allow the fast calculation of molecular dynamics trajectories [75,76].

The ADMP method extends to QM/MM processing of biological systems and to calculations of periodic systems using atomically centered functions [77]. Different systems are also actively studied using ADMP [78,79].

**Figure 13:**
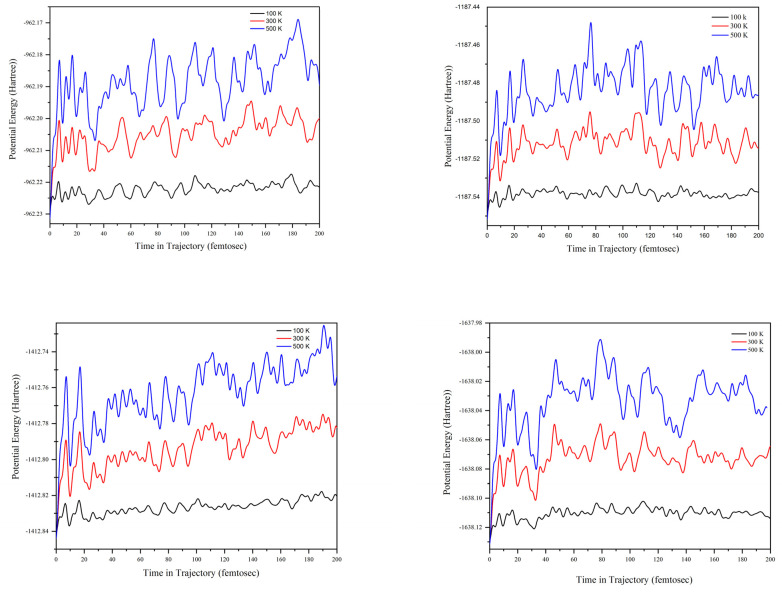
Relative potential energy trajectory curves for the ammonium sulfamate–urea system at different urea contents and different temperatures.

The relative potential energy trajectory curves for the ammonium sulfamate–urea system with different urea contents at different temperature are shown at Figure 13.

We carried out the ADMP calculation for the ammonium sulfamate–urea system with different component ratios at temperatures of 100, 300, and 500 °K. It can be seen in Figure 13 that, at all the investigated component ratios, the potential energy change (the curve amplitude) increases regularly with an increase in temperature from 100 to 500 °K. At 100 °K, the relative potential energy trajectory curves have the smallest amplitude. The intense peaks in the region of 0–20 fs are observed at the milestones of the investigated ratios, which increase significantly with the increasing temperature.

## 3. Experimental

### 3.1. Materials and Methods

Ammonium sulfamate and urea (Khimreaktivsnab LLC., Krasnoyarsk, Russia) were used. The reagents were preliminarily dried in an oven at 50 °C for 2 h.

The Fourier-transform infrared (FTIR) spectra were recorded on a Shimadzu IR Tracer-100 spectrometer (Japan) with a wavelength range of 400–4000 cm^−1^. Solid samples for the analysis were tablets in a KBr matrix (2-mg sample/1000-mg KBr).

The X-ray diffraction (XRD) study was carried out on a DRON-3 X-ray diffractometer (monochromatic Cu*K*_α_ radiation, λ = 0.154 nm) at a voltage of 30 kV and a current of 25 mA. The measurements were performed in the 2Θ Bragg angle range of 5.00–70.00 Θ.

The thermal analysis was carried using a NETZSCH STA 449 F1 Jupiter instrument in a corundum crucible at temperatures of 30–900 °C upon heating in an argon flow (the shielding and purge gas flow rates were 20 and 50 mL/min, respectively) at a rate of 10 °C/min. The measurement data were processed in the NETZSCH Proteus Thermal Analysis 5.1.0 software package supplied with the instrument.

The melting points were determined using an Electrothermal IA 9100 melting point apparatus.

### 3.2. Theoretical Calculation

The chemical potential was calculated using the formula

(2) $$\Delta \mu_{i}^{T}=-\Delta H_{m}*\left(1-\frac{T_{m}}{T_{i}}\right)$$

where $\Delta \mu_{i}^{T}$ is the chemical potential of component *i* at the eutectic temperature, *T_m_* is the melting point of a system, *T_i_* is the melting point of component *i*, and ∆H_*m*_ is the melting enthalpy of component *i* in a system.

Using the theory of real solutions [80], we calculated the effective interaction parameter for component *i* as

(3) $$\chi_{i}=-\frac{Ln\left(x_{i}\right)-\frac{\Delta \mu_{i}}{RT}}{{\left(1-x_{i}\right)}^{2}}$$

Using the additivity principle, we calculated the effective interaction parameter for the system as

χ = x_1_·χ_1_ + x_2_·χ_2_ (4)

### 3.3. Calculation Details

To optimize the geometrical parameters of the investigated system, including bond lengths and angles, the B3LYP/6-311++G(d, p) density functional theory (DFT) calculation was made using the Gaussian program [81]. The molecular structure was visualized in the GaussView software [82]. The noncovalent interactions were analyzed using the quantum theory of atoms in molecules (QTAIM) approach for studying the electron density at bond critical points (BCPs) implemented in the AIMall program [83]. In the Multiwfn wavefunction analysis package [84], the electron localization function (ELF) maps for studying the topology were built. Weak bond interactions, including especially hydrogen bonding, were determined using the reduced density gradient (RDG) study in the Multiwfn software and isosurfaces were visualized in the VMD software package [85]. The Frontier molecular orbitals (FMOs) were determined using the GaussView molecular visualization program to investigate the electronic properties, stability, and reactivity of the investigated compound. The molecular electrostatic potential (MEP) surfaces were mapped at the same level of theory to distinguish electrophilic and nucleophilic sites within the molecule. Atom-Centered Density Matrix Propagation (ADMP) calculations were carried out using the Gaussian 09 package program at the same level of theory.

## 4. Conclusions

For the first time, the ammonium sulfamate–urea binary system with different component ratios was studied comprehensively. It was shown with FTIR spectroscopy that this system remains stable at temperatures of up to 100 °C for up to 30 min without forming new chemical bonds. It was demonstrated using the X-ray diffraction analysis that temperatures below 100 °C and a time of 30 min barely affect the crystal structure of the system. The impact of temperature on the state of aggregation of the ammonium sulfamate–urea system with different component ratios was determined using the thermal analysis and melting point data. Theoretical methods were used to find the parameter of the effective interaction of the system, which indicates a strong attraction of molecules in it. The blue RDG contour between −0.05 and −0.03 a.u. pointed out the presence of a hydrogen bond, which was confirmed also by the effective interaction parameter χ. Using DFT methods, the molecular structures of the systems with different component ratios were optimized. The spectroscopy characteristics of the ammonium sulfamate–urea system with different component ratios were theoretically established. The ADMP calculation of the ammonium sulfamate–urea system at temperatures of 100, 300, and 500 K showed that, at all the investigated ratios, the potential energy (curve amplitude) change increases regularly with an increase in temperature from 100 to 500 K. The results obtained can help understand the physical chemistry of urea-containing binary systems.

## Figures and Tables

**Table 1:** Kinetic characteristics of thermal decomposition of substances.

Sample	Temperature Range, °C	Activation Energy (E_a_), kJ/mol	Preexponential Multiplier (A), s^−1^
U	162–247	97.0	1.9 × 10^9^
ASA	217–467	49.6	7.2 × 10^6^
ASA:U 1:1	175–256	54.7	3.0 × 10^5^
ASA:U 1:2	175–415	48.5	1.6 × 10^6^
ASA:U 1:3	160–395	45.5	2.3 × 10^6^
ASA:U 1:4	146–376	45.4	2.1 × 10^6^

**Table 2:** Experimental and theoretical melting points of the ammonium sulfamate–urea system.

Sample	Melting Point, °C
Urea (U)	133
Ammonium sulfamate (ASA)	131
ASA:U 1:1	84
ASA:U 1:2	87
ASA:U 1:3	90
ASA:U 1:4	96

**Table 3:** Temperature (°C) dependence of the phase state of the ammonium sulfamate–urea system.

Sample	Solid	Liquid	Gas
ASA:U 1:1	<83	84–175	>176
ASA:U 1:2	<86	87–175	>175
ASA:U 1:3	<89	90–165	>165
ASA:U 1:4	<95	96–146	>146

**Table 4:** Chemical potentials of the ammonium sulfamate–urea system.

Sample	Δμ_i_^T^ (ASA), kJ/mol	Δμ_i_^T^ (U), kJ/mol
ASA:U 1:1	−2.06	−2.06
ASA:U 1:2	−3.32	−1.20
ASA:U 1:3	−4.18	−0.87
ASA:U 1:4	−4.94	−0.68

**Table 5:** Effective interaction parameters of the ammonium sulfamate–urea system.

Sample	χ (ASA)	χ (U)	χ (ASA:U)
ASA:U 1:1	−2.77	−2.77	−2.77
ASA:U 1:2	−2.47	−3.67	−3.28
ASA:U 1:3	−2.46	−4.60	−4.06
ASA:U 1:4	−2.51	−5.57	v4.96

**Table 6:** Physiochemical descriptors calculated using the FMO energies.

Parameter (eV)	*N* = 1	*N* = 2	*N* = 3	*N* = 4
*E*_HOMO_	−7.1300	−7.1600	−7.0700	−7.0400
*E*_LUMO_	−0.7100	−0.6600	−0.9000	−1.2600
Energy gap	6.4200	6.5000	6.1700	5.7800
Ionization potential	7.1300	7.1600	7.0700	7.0400
Electron affinity	0.7100	0.6600	0.9000	1.2600
Electronegativity	3.9200	3.9100	3.9850	4.1500
Chemical potential	−3.9200	−3.9100	−3.9850	−4.1500
Chemical hardness	3.2100	3.2500	3.0850	2.8900
Chemical softness	0.3115	0.3077	0.3241	0.3460
Global electrophilicity index	2.3935	2.3520	2.5738	2.9797
Maximum charge transfer index	1.2212	1.2031	1.2917	1.4360
Nucleophilicity index	0.4178	0.4252	0.3885	0.3356
Optical softness	0.1558	0.1538	0.1621	0.1730

**Table 7:** Thermodynamic parameters of ammonium sulfamate–urea systems with different numbers of urea molecules at 298.150 K calculated in the DFT/B3LYP/6-311++G(d, p) basis set.

Parameter	*N* = 1	*N* = 2	*N* = 3	*N* = 4
E(RB3LYP) (a.u.)	−962.23145	−1187.5518	−1412.844	−1638.1316
Dipole Moment (Debye)	7.6544727	5.3019744	7.6456568	11.870975
Polarizability (a.u.)	86.585041	121.01455	155.60431	188.86192
Hyperpolarizability (a.u)	106.25774	141.08799	173.68145	248.69445
Electronic Energy (EE) (a.u.)	−962.23145	−1187.5518	−1412.844	−1638.1316
Zero-Point Energy Correction (a.u.)	0.15438	0.223065	0.289022	0.354993
Thermal Energy Correction (a.u.)	0.168616	0.242165	0.313932	0.385602
Thermal Enthalpy Correction (a.u.)	0.16956	0.243109	0.314876	0.386546
Thermal Free Energy Correction (a.u.)	0.11061	0.174478	0.230917	0.291085
EE + Zero-Point Energy (a.u.)	−962.07707	−1187.3288	−1412.555	−1637.7766
EE + Thermal Energy Correction (a.u.)	−962.06283	−1187.3097	−1412.53	−1637.746
EE + Thermal Enthalpy Correction (a.u.)	−962.06189	−1187.3087	−1412.5291	−1637.745
EE + Thermal Free Energy Correction (a.u.)	−962.12084	−1187.3773	−1412.6131	−1637.8405
E (Thermal) (kcal/mol)	105.808	151.961	196.995	241.969
Heat Capacity (Cv) (cal/mol-kelvin)	47.822	66.847	86.688	106.05
Entropy (S) (cal/mol-kelvin)	124.071	144.447	176.708	200.914

## Data Availability

Not applicable.

## Supplementary Materials

The following supporting information can be downloaded at: https://www.mdpi.com/article/10.3390/molecules28020470/s1, Table S1. AIM topological parameters for the ammonium sulfamate–urea system.
