# Supplementary material for: Comprehensive Study of the Ammonium Sulfamate–Urea Binary System
Source: Molecules. 2023 Jan 4;28(2):470. doi: 10.3390/molecules28020470 (PMC9861415; doi:10.3390/molecules28020470)
Supplement: Supplementary file 1 [file molecules-28-00470-s001.zip › molecules-2130904-supplementary.pdf]

Supplementary Materials

Comprehensive Study of the Ammonium Sulfamate–Urea Binary System

Aleksandr S. Kazachenko [1,2,3,*], Noureddine Issaoui [4], Olga Yu. Fetisova [2], Yaroslava D. Berezhnaya [2], Omar M. Al-DOSSARY [5], Feride Akman [6], Naveen Kumar [7], Leda G. Bousiakou [8], Anna S. Kazachenko [1], Vladislav A. Ionin [1,2], Evgeniy V. Elsuf'ev [1,2] and Angelina V. Miroshnikova [1,2]

[1] Department of Organic and Analytical Chemistry, Siberian Federal University, pr. Svobodny 79, 660041 Krasnoyarsk, Russia
[2] Institute of Chemistry and Chemical Technology, Krasnoyarsk Scientific Center, Siberian Branch, Russian Academy of Sciences, Akademgorodok 50, Bld. 24, 660036 Krasnoyarsk, Russia
[3] Department of Biological Chemistry with Courses in Medical, Pharmaceutical and Toxicological Chemistry, Krasnoyarsk State Medical University, St. Partizan Zheleznyak, Bld. 1, 660022 Krasnoyarsk, Russia
[4] Laboratory of Quantum and Statistical Physics (LR18ES18), Faculty of Sciences, University of Monastir, Monastir 5079, Tunisia
[5] Department of Physics and Astronomy, College of Science, King Saud University, P.O. Box 2455, Riyadh 11451, Saudi Arabia
[6] Vocational School of Food, Agriculture and Livestock, University of Bingöl, Bingöl 12000, Turkey
[7] Department of Chemistry, Maharshi Dayanand University, Rohtak 124001, India
[8] IMD Laboratories Co., R&D Section, Lefkippos Technology Park, NCSR Demokritos, P.O. Box 60037, 15130 Athens, Greece
* Correspondence: kazachenko.as@icct.krasn.ru or askazachenko@sfu-kras.ru

Table S1. AIM topological parameters for the ammonium sulfamate–urea system

BCP	ϱ (r)	∇²ϱ (r)	V	G	K	L	E inter	Ellipticity
***N* = 1**								
H5 … H20	+0.008945	3.99476e-18	-0.006446	+0.007398	-0.000952	-0.008350	-8.3798	+0.299088
H12… N15	+0.019059	1.60802e-17	-0.013591	+0.013513	+0.000078	-0.013435	-17.6683	+0.046143
O4… H18	+0.018597	3.89271e-17	-0.015506	+0.015403	+0.000103	-0.015300	-20.1578	+0.053690
***N* = 2**								
H18… O24	+0.012353	+0.044206	-0.009381	+0.010216	-0.000835	-0.011051	-12.1953	+0.154589
O3… N6	+0.011597	+0.045085	-0.008572	+0.009922	-0.001350	-0.011271	-11.1436	+1.286594

O4 … H28	+0.016922	+0.056266	-0.013992	+0.014029	-0.000037	-0.014067	-18.1896	+0.105453
O4… H26	+0.019081	+0.065762	-0.016067	+0.016253	-0.000187	-0.016440	-20.8871	+0.067441
H20 …O24	+0.020503	+0.066684	-0.016913	+0.016792	+0.000121	-0.016671	-21.9869	+0.029040
O3… H17	+0.023746	+0.077578	-0.02015	+0.019772	+0.000378	-0.019394	-26.195	+0.025699
O5 … H7	+0.045372	+0.138790	-0.038308	+0.036503	+0.001805	-0.034697	-49.8004	+0.042730
H10 …O16	+0.058894	+0.179439	-0.051324	+0.048092	+0.003232	-0.044860	-66.7212	+0.024374
***N* = 3**								
O5 … H7	+0.043568	+0.133744	-0.036732	+0.035084	+0.001648	-0.033436	-47.7516	+0.040146
O3 … H26	+0.021569	+0.068362	-0.018222	+0.017657	+0.000566	-0.017091	-23.6886	+0.055882
O3… H17	+0.021331	+0.068850	-0.018051	+0.017632	+0.000419	-0.017212	-23.4663	+0.030460
H20 …O32	+0.020041	+0.066144	-0.01656	+0.016548	+0.000012	-0.016536	-21.528	+0.027807
O4 … H34	+0.018483	+0.063026	-0.015536	+0.015646	-0.000110	-0.015757	-20.1968	+0.042475
O4 … H28	+0.015113	+0.049144	-0.012152	+0.012219	-0.000067	-0.012286	-15.7976	+0.039806
H11… N23	+0.015596	+0.045847	-0.010775	+0.011118	-0.000343	-0.011462	-14.0075	+0.092361
H18… O32	+0.013094	+0.046474	-0.010078	+0.010848	-0.000770	-0.011619	-13.1014	+0.126377
O4 … H36	+0.012306	+0.042990	-0.009754	+0.010251	-0.000497	-0.010748	-12.6802	+0.126196
O3… N6	+0.009878	+0.038456	-0.007148	+0.008381	-0.001233	-0.009614	-9.2924	+1.453816
***N* = 4**								
O3 … H8	+0.043805	+0.135070	-0.037038	+0.035403	+0.001635	-0.033767	-48.1494	+0.044126
O5… H7	+0.016054	+0.057459	-0.012874	+0.013619	-0.000745	-0.014365	-16.7362	+0.258061
O3 … H18	+0.010971	+0.039536	-0.008662	+0.009273	-0.000611	-0.009884	-11.2606	+0.108243
N15… H28	+0.024520	+0.065089	-0.017881	+0.017077	+0.000804	-0.016272	-23.2453	+0.009599
O4… H20	+0.018347	+0.059524	-0.0153	+0.015091	+0.000210	-0.014881	-19.89	+0.053887
O5 … H26	+0.013941	+0.048069	-0.011433	+0.011725	-0.000292	-0.012017	-14.8629	+0.061935
H10… N23	+0.045469	+0.108990	-0.034375	+0.030811	+0.003564	-0.027247	-44.6875	+0.019004
N23… N39	+0.003571	+0.010232	-0.002101	+0.002330	-0.000228	-0.002558	-2.7313	+0.891477
O4… H34	+0.011830	+0.041644	-0.00937	+0.009890	-0.000521	-0.010411	-12.181	+0.088359
H25 …O32	+0.009037	+0.033363	-0.006404	+0.007372	-0.000968	-0.008341	-8.3252	+0.366027
O5 … H36	+0.010541	+0.037746	-0.008069	+0.008753	-0.000684	-0.009437	-10.4897	+0.148248
O24… N37	+0.004896	+0.016307	-0.002975	+0.003526	-0.000551	-0.004077	-3.8675	+0.967446

O32 …H41	+0.020743	+0.065453	-0.016975	+0.016669	+0.000306	-0.016363	-22.0675	+0.068728
O16 …H43	+0.014767	+0.047366	-0.011629	+0.011735	-0.000106	-0.011841	-15.1177	+0.057771
O24 …N39	+0.003930	+0.013002	-0.002325	+0.002788	-0.000463	-0.003250	-3.0225	+0.667436
